# Clinical features, course and treatment of methamphetamine-induced psychosis in psychiatric inpatients

**DOI:** 10.1186/s12888-016-0745-5

**Published:** 2016-02-25

**Authors:** Homa Zarrabi, Mohammadrasoul Khalkhali, Azam Hamidi, Reza Ahmadi, Maryam Zavarmousavi

**Affiliations:** Department of Psychiatry, Shafa University Hospital, Guilan University of Medical Sciences, Panzdah Khordad Ave., 4165863795 Rasht, Iran

**Keywords:** Methamphetamine, Psychotic disorders, Inpatients, Violence

## Abstract

**Background:**

Over the past few years, methamphetamine-induced psychosis (MIP) has increased in Iran, accounting for a significant percentage of psychiatry hospital admissions. The present study was conducted with an aim to investigate clinical symptoms, and course and treatment methods of MIP inpatients in Shafa Psychiatry Hospital in northern Iran.

**Methods:**

Participants were 152 MIP inpatients. Brief Psychiatric Rating Scale (BPRS) subscales of suspiciousness, unusual thought content; hallucinations and hostility were used to measure psychiatric symptoms. Data regarding suicide and homicide and violence were also obtained through interviews with the inpatients and their family. Based on their lengths of recovery time, the inpatients were categorized into 3 clinical groups. These inpatients received their usual treatments and were monitored for their psychiatric symptoms and clinical course of illness. The data were analyzed by descriptive statistics.

**Results:**

The most frequent psychiatric symptoms were violence (75.6 %), intimate partner violence (61.2 %), delusions of persecution (85.5 %), delusions of reference (38.5 %), delusions of grandiosity (32.9 %), delusions of infidelity (30.2 %), auditory hallucinations (51.3 %), visual hallucinations (18.4 %), suicidal thoughts (14.5 %), homicidal thoughts (3.9 %), suicide attempts (10.5 %) and homicide attempts (0.7 %). Recovery from psychotic symptoms in 31.6 % of the inpatients took more than one month. 46.1% of the inpatients were treated with Risperidone and 37.5 % with Olanzapine. Persecutory delusion and auditory hallucination were the most frequent persistent psychotic symptoms. 20.8 % of the inpatients with duration of psychosis more than one month were treated with electroconvulsive therapy (ECT) along with antipsychotics.

**Conclusion:**

All forms of violence are highly frequent in MIP inpatients. Our finding agrees with many other studies suggesting that recovery from MIP can take more than a month. Initial promising findings were found regarding the efficacy of Electroconvulsive therapy in MIP patients.

## Background

Acute psychotic disorder is a common phenomenon in the abuse of amphetamines and methamphetamine, and one of the most important reasons for referring to and hospitalization in emergency or psychiatric wards [1–3]. The psychotic symptoms in acute intoxication are persecutory, influence and control delusions, auditory, visual and tactile hallucinations, and a tendency toward violence [4–6]. In certain cases, even after the symptoms of intoxication disappear and the substance abuse is not repeated, these symptoms are seen again in prolonged manners. These symptoms cannot be differentiated from other psychotic symptoms of schizophrenia spectrum disorders [4–9].

Acute psychosis induced by amphetamines is limited to a maximum period of 4 to 5 days after the intoxication, and appears to resolve with abstinence; although the recovery may be incomplete [9]. Japan is the origin of methamphetamine. This country has experienced several major epidemics of the substance. Japanese psychiatrists believe that stimulants-induced psychosis can be divided into different clinical groups. The first group is the transient psychosis group in which the incidence and duration of symptoms are limited to a maximum period of 4 to 5 days after the intoxication, which can be seen in the withdrawal or intoxication periods. In the second group, psychotic symptoms can be resolved in less than a month [7, 8]. In the third group, symptoms could be of much longer duration of up to several months, or of several years [7–10]. Some experts believe that 5 to 10 % of the MIP patients may not fully recover from their psychotic symptoms even after a long time [7, 8]. The studies recently carried out as well as those in Iran have provided strong clinical evidence to support such a classification [6, 7, 11, 12]. In Yui K et al. [7] study, 64 % of patients recovered from their symptoms within ten days, and 82 % gained full recovery within a month, and in 18 %, psychosis persisted more than a month. In a previous study in Iran, 8.75 % of the cases with MIP had symptoms that continued more than a month [6]. In the 3 major epidemics in Japan, a significant number of patients still had the psychotic symptoms after a month [7, 8]. Some researchers believe that MIP can imitate both positive and negative symptoms of schizophrenia, and some consider it, a short-term psychosis with positive symptoms. It is difficult to distinguish the Japanese persistent psychosis from a primary psychosis like schizophrenia triggered by the use of amphetamines [9, 12].

There are still many unanswered questions regarding clinical characteristics of MIP. Unfortunately, Iran has experienced an epidemic of methamphetamine use in recent years, which has provided an opportunity to study clinical symptoms, and course and treatment methods of MIP inpatients.

## Methods

This cross sectional study was conducted at Shafa Psychiatry Hospital, the only psychiatric hospital, referral and psychiatric emergencies center in Rasht, the capital of Guilan Province, with a population of 2.5 million, in northern Iran, from August 2013 to August 2014.

Participants were 152 MIP inpatients who were admitted to Shafa Hospital due to clinically significant symptoms of hostility, grandiosity or suspiciousness according to a score of 5 or above, and hallucinations, unusual thought content or suicidality according to a score of 6 or above on the Brief Psychiatric Rating Scale (BPRS) [13]. The hostility in the month preceding admission was considered violence. The urine screening tests for detecting amphetamines, morphine and cannabis were performed at the time of admission in the emergency ward. The patients were interviewed about their drug and substance abuse and dependency. The family observations of intoxication periods and substance related behaviors in the preceding month of admission were obtained in separate face-to-face interviews and were used to determine the abuse of any probable substances. The medical records were used to determine schizophrenia, bipolar disorder, antisocial and borderline personality disorder. If positive urine tests or any history of abuse of alcohol, cannabis or morphine in the preceding month of admission was detected or if the patients were diagnosed with any of the above-mentioned primary psychiatric disorders, they were excluded from the study. Fixed doses of methadone were accepted. All of the patients of this study had positive urine tests for methamphetamine. A psychiatrist evaluated the patients and concluded that they met the DSMIV-TR diagnostic criteria for amphetamine-like use disorders (dependence; 304.40 or abuse; 305.70) and amphetamine-like induced psychotic disorders (292.11, 292.12).

BPRS was administered according to the guidelines provided by Lukoff [13] at admission time and then twice weekly to assess psychiatric symptoms and to monitor the patients’ improvement. After the patients were admitted to the psychiatric wards and the primary evaluations were completed, treatment was undertaken by certified psychiatrists. The inpatients were monitored for primary psychiatric symptoms and clinical course of treatment, and they received usual evaluation and treatment for psychotic disorders. There was no intervention or compromise regarding treatment methods, whether medicinal or otherwise. In order to be able to compare the results of this study with other studies, considering the patterns of recovery of patients in the previous studies, we divided the patients in into 3 clinical groups:

*Group 1* (full recovery equal or less than one week), *Group 2* (full recovery more than one week but equal or less than one month), and *Group 3* (full recovery more than one month). Considering that there is no global agreement and clear definition for the terms “transient” and “persistent”, we preferred to use numbered groups in this paper. Achieving a score of 3 or lower in any of the mentioned subscales was the criteria for the inpatient’s recovery in that particular subscale, and full recovery was determined by recovery in all of the subscales of BPRS. BPRS is a tool developed for assessing the degree of severity of psychiatric symptoms and is widely used in clinical and research assessments, and enjoys a high degree of inter-rater reliability when used by skilled clinical specialists. Certified psychiatrists and an experienced clinical psychologist who had been trained in all aspects of the interviewing skills, conducted interviews.

Data regarding suicide, homicide and violence were also obtained from interviews with the inpatients and their family by certified psychiatrists and a clinical psychologist, as well as from other referral sources and, if necessary, from local investigations by social workers. We asked the family members whether the patients had assaulted them with no harm repeatedly, for example, slapped or pushed them, whether the patients had severely destroyed the properties in the house or outside, for example, knocked over furniture, broken windows, and burned things, or whether the patients had assaulted them with definite possibility of harming or with actual harm, for example, assaulted them with a hammer or a knife? In this study, we only considered sever physical violence.

While hospitalized, we monitored the inpatients closely, regarding abuse of illicit drugs and substances every 3 days, using clinical evaluation and urine screening tests. Inpatients who did not continue with the treatment or, for any reason, tested positive for any substance abuse during the hospitalization period were excluded from this study.

The written consents were obtained both from the patient and from a responsible (legally authorized) family member in each case. This study received ethics approval from the Research Ethics Committee of Guilan University of Medical Sciences (IR.GUMS.REC.1394.253). The data were analyzed by SPSS-15. The Chi-Square test was used for statistical analysis, and values less than 0.05 were considered meaningful.

## Results

Out of 2600 Shafa Hospital admissions from August 2013 to August 2014, 1173 patients had positive urine tests for methamphetamine. Eventually 152 MIP inpatients formed the sample of this study and the others were excluded due to exclusion criteria. Table 1 shows the demographic characteristics of our study. The average age of the population was 35.7 (8.07) years of age, with the highest frequency in the 30–40 year age group.

**Table 1:** Demographic characteristic of 152 MIP inpatients

Variables					
Age (year)	N (%)	19–30 33(21.7)	31–40 75(49.3)	41–50 34(22.4)	>50 10(6.6)
Gender	N (%)	Male 139 (94.1)	Female 13 (8.6)		
Marital status	N (%)	Married 49 (32.2)	Single 103 (67.8)		
Education	N (%)	Illiterate 12 (7.8)	<High school 119 (78.3)	High school 20 (13.2)	University graduate 1 (0.7)

Table 2 shows the clinical characteristics of our study. 115 of the inpatients (75.6 %) had at least one form of violent behavior in the past month. Of the 49 married inpatients, 30 married inpatients (61.2 %) displayed intimate partner violence. It must be noted that a person may have multiple aggressive behaviors; therefore, the statistics yielded various answers. 146 of the inpatients (96.1 %) had delusions or hallucinations. 6 of the patients (3.9%) showed no symptoms of delusions or hallucinations at the time of admission, but they were hospitalized for disorganized behaviors. No statistically meaningful relation was found between psychotic symptoms (delusions and hallucinations) and different forms of violence. The only significant statistical difference was found between intimate partner violence and delusions of reference (*p* < 001). Persecutory delusion and auditory hallucination were the most frequent persistent psychotic symptoms. The frequencies of grandiosity delusion (*p* < 0.01) and auditory hallucination (*p* < 0.05) were significantly different in clinical groups (Table 2).

**Table 2:** The frequency of psychiatric symptoms in 152 MIP inpatients

	^a^Group1N (%)	^b^Group 2 N (%)	^c^Group3 N (%)	Total N (%)	*P
Delusions/hallucinations					
Persecutory	16(94.1)	75(86.2)	39(81.3)	130(85.5)	0.469
Reference	9(52.9)	29(33.3)	21(43.8)	59(38.8)	0.221
Grandiosity	1(5.9)	27(31)	22(45.8)	50(32.9)	0.008
Infidelity	6(35.3)	26(29.9)	14(29.2)	46(30.2)	0.862
Thought broadcasting	1(5.9)	0(0)	1(2.1)	2(1.3)	0.083
Thought insertion	0(0)	0(0)	1(2.1)	1(0.7)	0.428
Thought withdrawal	1(5.9)	0(0)	1(2.1)	2(1.3)	0.083
Somatic delusion	1(5.9)	2(2.3)	4(8.3)	7(4.6)	0.196
Auditory hallucinations	11(64.7)	37(42.5)	30(62.5)	78(51.3)	0.041
Visual hallucinations	2(11.8)	14(16.1)	12(25)	28(18.4)	0.349
Tactile hallucinations	1(5.9)	0(0)	1(2.1)	2(1.3)	0.083
Violence					
IPV**	4(23.5)	21(24.1)	6(12.5)	31(20.4)	0.241
Family	9(52.9)	50(57.5)	25(52.1)	84(55.3)	0.839
Others	8(47.1)	64(73.6)	33(68.8)	91(69.1)	0.097
Suicide /Homicide					
Suicidal thought	2(11.8)	9(10.3)	11(22.9)	22(14.5)	0.142
Suicide attempt	2(11.8)	8(9.2)	6(12.5)	16(10.5)	0.738
Homicidal thought	0(0)	4(4.6)	2(4.2)	6(3.9)	1.000
Homicide attempt	0(0)	0(0)	1(2.1)	1(0.7)	0.428

a: Full recovery equal or less than one week

b: Full recovery more than one week but equal or less than one month

c: Full recovery more than one month

*Pearson chi square and for table with at least 1 cell expected count less than 5, Fisher’s exact test. *p* <0.05 considered significant

** Intimate partner violence

Table 3 shows the relation between the treatment methods and clinical groups. The patients were treated with antipsychotics, supportive therapy and ECT (Electroconvulsive Therapy). Serotonin- Dopamine Antagonists, especially Risperidone, (46.1 %) and Olanzapine (37.5 %) were the most frequently prescribed antipsychotics. The use of typical antipsychotics was significantly low. Table 4 shows the frequency of the use of antipsychotic drugs in different clinical groups.

**Table 3:** The frequency of prescription of antipsychotics in treating MIP inpatients

		Risperidone	Olanzapine	Quetiapine	Aripiprazole	Haloperidol	Perphenazine
^a^ Group 1	N (%)	9 (65.2)	5 (31.3)	1 (6.25)	0 (0)	1 (6.25)	0 (0)
^b^ Group 2	N (%)	38 (44.2)	38 (44.2)	7 (8.2)	3 (3.4)	0 (0)	0 (0)
^c^ Group 3	N (%)	23 (48)	13 (27.1)	3 (6.2)	8 (16.6)	0 (0)	1 (2.4)

a: full recovery equal or less than one week

b: full recovery more than one week but equal or less than one month

c: full recovery more than one month

**Table 4:** The frequency of methods used to treat 152 MIP inpatients

		Supportive therapy	Antipsychotics	ECT*	Antipsychotics and ECT
^a^ Group 1	N (%)	1 (5.9)	16 (94.1)	0 (0)	0 (0)
^b^ Group 2	N (%)	1 (1.1)	70 (80.5)	1 (1.1)	15 (17.2)
^c^ Group 3	N (%)	0 (0)	37 (77.1)	1 (2.1)	10 (20.8)

a: full recovery equal or less than one week

b: full recovery more than one week but equal or less than one month

c: full recovery more than one month

*ECT Electroconvulsive therapy

## Discussion

Methamphetamine is the second most frequently abused illicit drug in Iran [11, 14] and many individuals who referred to psychiatric emergency wards abused methamphetamine [1]. Among those admitted, other substances usually accompany methamphetamine abuse, which makes it difficult to relate psychiatric symptoms to one particular substance. Due to the exclusion criteria in the present study, it may be claimed that this study can provide a more accurate account of psychiatric symptoms connected with methamphetamine abuse [3, 5].

Like other studies, persecutory delusion and auditory hallucination were the most frequent psychiatric symptoms, and almost all inpatients had experienced a few psychiatric symptoms. These symptoms, along with delusion of reference, grandiosity delusion and delusions of infidelity to the spouse can hardly be differentiated from other primary psychotic disorders such as schizophrenia [15–17]. In addition, delusion of grandiosity and aggression can be frequently seen in manic episodes of bipolar disorder.

In comparison with the previous study conducted in Iran, the average age of the population was a little higher (35.7 vs. 30.44), and an approximately two-fold increase in the number of female inpatients (8.6 % vs. 4.5 %) was cited [6]. The percentage of improvement of the symptoms in the first week was the same, but the persistence of the symptoms over one month was significantly higher in our study (31.6 % vs. 8.75 %). This means that methamphetamine takes a huge toll on the health care system. On the one hand, not only has there been no decrease in the rate of mood disorders and schizophrenia and other psychotic disorders and substance-related disorders, but the health care system is also faced with a phenomenon that has caused a majority of beds in psychiatric hospitals and emergency wards to be occupied.

The findings of our study are more in line with the Japanese studies which reported the rates of persistent psychosis in the 3 epidemics in Japan as 23, 18 and 41 respectively [6]. This may of course have been partly due to our sampling methods, since the probability of psychosis induced by non-amphetamine substances and primary psychotic disorders in our group have been reduced to a minimum. It is also probable that our patients were exposed to the substance for a longer period or a larger dose of the substance.

Intimate Partner Violence (IPV) is a form of domestic violence in which the spouse will be a target for physical, and psychological, economic and sexual harm [18]. Regarding the particular symptoms of this disorder such as paranoia, and delusions of infidelity, it is assumed that these symptoms can make for intimate partner violence.

There is a high rate of IPV in certain groups of the society, including substance abusers, especially methamphetamine abusers [19–23]. However, in our study, only sever physical violence was studied, which calls for further research. In the present study, three-thirds of those sampled displayed at least one form of violence (toward the spouse, family, and society), and 61.2 % of them met the criteria for severe physical IPV. Although our descriptive study may not be able to demonstrate a causal relationship between methamphetamine abuse and violent behavior, due to exclusion criteria of our study, it can be concluded that the violent behaviors only emerge after having abused methamphetamine. McKetin et al. [17] have reported that there is a clear increase in violent behaviors among methamphetamine abusers compared to when they do not abuse the substance. The findings of the present study indicate that there is a significantly high rate of physical violence in MIP inpatients. Special attention must be paid to the spouses and children of the married patients, and parents and siblings of the single patients who spend many days with the methamphetamine-addicted patients, and consequences of violence such as depression, anxiety and post-traumatic stress disorder should be explored [24, 25]. Unfortunately, admissions in the psychiatric hospitals in Iran mainly focus on removing psychiatric symptoms until the patient leaves the hospital, and do not much focus on the impact of the illness on the family members. It seems that the facilities available now may not be suitable or efficient enough.

Persistent psychosis has only been mentioned in the published works of East Asian researchers. In recent clinical studies, such as those done by Grelotti DJ et al. [12], more attention has been paid to studies in the East Asia. These studies have accepted that persistent psychosis can still emerge after methamphetamine abuse [12]. Published works of Iranian researchers in recent years confirm such a claim too [11, 14]. There is a growing belief that adequate amounts and the period of methamphetamine abuse, without any special psychiatric history, can lead to MIP due to damage caused to Dopaminergic, Serotoninergic, and Noradrenergic cells [26–29]. Medhus et al. [30] found that among the patients with MIP, one third was diagnosed with schizophrenia during a 6-year follow up. In a similar study in Thailand, 38 % of MIP cases were given a diagnosis of schizophrenia, 6-years after their hospitalization [31]. Niemi-Pyntarri et al. [32] reported that the 8-year cumulative risk to receive a schizophrenia spectrum diagnosis was 30 % for MIP patients. This has been argued for using MIP as a model for primary psychotic disorders and schizophrenia [33].

Despite the widespread abuse of methamphetamine and the related psychosis, there is no structured guideline for treating the MIP. The number of clinical trials has been too low to help formulate a comprehensive manual to be used by the therapists [34–38]. In the present study, Risperidone and Olanzapine had the highest frequency of use of all antipsychotics, which can partly be the result of the current clinical trials indicating the positive effects of Risperidone in treating MIP [39]. These drugs are effective in restoring the weight, which patients considerably lost because of methamphetamine-induced appetite loss and hyperactivity. Almost all the patients in group1 received antipsychotic drugs. The overall approach is supportive treatment followed by the application of benzodiazepines in order to reduce restlessness and agitation [40]. Findings indicate that our therapists prefer the application of antipsychotics. The reasons may be the possibility of better control of violent behaviors, the therapist’s expectation that the patients will not be classified in group 1, or the number of our hospital beds that is not proportionate to the number of patients. The one-week period within which the patients are not given antipsychotics can extend the length of hospitalization. In this approach, the clinician is not able to determine whether the patient responded to antipsychotics, or they recovered on their own, that can increase the risk of inappropriate use of antipsychotics.

The recovery in 70% of the patients happened in less than a month, but at the end of the one month, at least 30% of the patients still had the symptoms. The application of ECT in group 2 aimed simply at controlling severe aggression and violent behaviors, thoughts of suicide and homicide. The ECT was applied for group 3, only when clinicians still detected persistent psychotic symptoms with no response to antipsychotics. The type of prescribed medication did not change during treatment with ECT in group 3. After 6 to 9 sessions of ECT, symptoms began to disappear. ECT helped improve psychotic symptoms alone or in combination with medication in nonresponsive-to-antipsychotic cases.

We did not come across any report of the application or effectiveness of ECT in treating MIP. It might seem that the application of ECT could be rather restricted in other countries. We found the only report of the effectiveness of ECT in treating MIP in a case study by Grelotti et al. [12]. Another reason might be that psychotic disorders exceeding more than one month, according to the American Psychiatric Association diagnostic criteria, are definitely considered primary psychiatric disorders [30–33],and therefore, the application of ECT in this case is allowed and effective. This reasoning rules out any such thing as persistent psychosis in the literature, let alone any treatment for it.

About 10% of the population in this study had suicidal thoughts one month prior to admission. Given the aggressive nature unique to these patients, in a hasty judgment, these patients are considered a source of aggression and hazard to other people. Additionally, compared to other psychiatric symptoms, suicide and self-aggression receive less attention. Methamphetamine abuse, in particular, has been followed by suicidal behaviors as well as fatal suicide attempts [41–47]. In blood and urine samples for toxicological analysis of suicide victims, alcohol and methamphetamine amounts were found to be significantly high [44]. In psychiatric emergency wards, the methamphetamine abusers are more likely to have experienced suicide attempts compared to other groups of inpatients [48].

Marshall and Werb [49] considered suicide and methamphetamine overdose as the main cause of death among adolescents and young adults abusing methamphetamine. It seems that the psychiatric symptoms of methamphetamine abusers along with depression may have contributed to this tendency [44, 45]. Many of participants in methamphetamine-related treatment programs suffer from depression [45, 46, 49]. The admissions in hospitals or the sudden intervals of substance withdrawals can be followed by depression and serious thoughts of suicide [50–55]. Thoughts of suspicion and constant thoughts of infidelity toward the spouse and feelings of fear and confusion over being always stalked can cause such feelings of helplessness in the patient of which, they begin to believe; only death can get them relieved. In addition, in the periods of intoxication, an increase in substance-related agitation and visual hallucinations were followed by a rise in the risk of suicide attempts [43, 44].

Homicide is the endpoint of violent behavior. Physical violent behavior can easily turn into homicide. Certain homicide attempts might result from the individual’s violent behavior and not prior intentions, or might follow other dominant thoughts of homicide. In a study, the risk of homicide by a methamphetamine abuser was estimated to be 9 times that by the control group. Methamphetamine seems to be the only substance with such a strong link with homicide [22].

There are some limitations for this study. First, this study mainly focused on recovery from the positive symptoms. What we expect to see in the long-term clinical course are spontaneous relapses and negative psychotic symptoms, which could be induced by neurotoxic and degenerative effects of the substance on nerve pathways. Second, only severe physical violence has been taken into account and other important types of violence have not been considered. Third, this study was descriptive with a local focus; therefore, further analytical research on other forms of violence with a national focus seems necessary. Fourth, although we have a remarkable number of psychiatrists and mental health professionals and trained general practitioners in mental health system in Guilan, but we do not have any structured program for detecting people in early stages of developing psychosis or schizophrenia. It is not clear whether methamphetamine acts as a trigger for the first episode of psychosis in vulnerable people, or whether it starts an independent neuropathologic process.

## Conclusion

The violence in these patients is significant and highly frequent. The frequency of persistent psychosis is 3 times the previous study in Iran, and closer to the statistics of the methamphetamine epidemics in Japan, indicating that the recovery time from psychotic symptoms is becoming longer in Iran, which agrees with many other studies suggesting that recovery from MIP can take more than a month. There were initial promising findings regarding the efficacy of treatment with electroconvulsive therapy, especially in treating persistent psychosis or reducing violent behaviors. In summary, the data present in this study can act as a motive to draw more attention to the harmful aspects of methamphetamine abuse in the Iranian society, especially regarding psychological health and social crimes. The results of this study are a serious warning, which calls for attention from psychiatrists and judicial authorities in devising proper preventive measures.

## Abbreviations

IPV: Intimate partner violence
MIP: Methamphetamine induced psychosis
ECT: Electroconvulsive therapy
BPRS: Brief psychiatric rating scale
DSMIV-TR: Diagnostic and statistical manual of mental disorders. 4th edition. Text revision
